# Step-by-Step Minimally Invasive Aortic Valve Replacement: the RAT Approach

**DOI:** 10.21470/1678-9741-2020-0393

**Published:** 2021

**Authors:** Alina Zubarevich, Konstantin Zhigalov, Bastian Schmack, Arian Arjomani Rad, Robert Vardanyan, Daniel Wendt, Arjang Ruhparwar, Alexander Weymann

**Affiliations:** 1 Department of Thoracic and Cardiovascular Surgery, West German Heart and Vascular Center, University of Duisburg-Essen, Essen, Germany.; 2 Department of Medicine, Faculty of Medicine, Imperial College London, Great Britain.

**Keywords:** Transcatheter Aortic Valve Replacement, Aortic Valve, Thoracotomy, Surgical Wound Infection, Frail Elderly, Heart Valve Prosthesis

## Abstract

In the growing era of transcatheter aortic valve implantation, it is crucial to develop minimally invasive surgical techniques. These methods enable easier recovery from surgical trauma, especially in elderly and frail patients. Minimally invasive aortic valve replacement (MIAVR) is frequently performed via upper hemisternotomy. We describe MIAVR via right anterior thoracotomy, which is associated with less trauma, rapid mobilization, lower blood transfusion rates, and lower risk of postoperative wound infections. As minimally invasive procedures tend to take longer operative times, we suggest using rapid-deployment valve prostheses to overcome this limitation. This description focuses on the technical aspects and preoperative assessment.

**Table:** 

Abbreviations, acronyms & symbols	
CPB	= Cardiopulmonary bypass
CT	= Computer tomography
ICU	= Intensive care unit
MIAVR	= Minimally invasive aortic valve replacement
RAT	= Right anterior thoracotomy
TAVI	= Transcatheter aortic valve implantation
TEE	= Transesophageal echocardiography

Since its first description in 1996^[1]^, minimally invasive aortic valve replacement (MIAVR) has been gaining popularity in cardiothoracic surgery. Thanks to its minimally invasive nature and low surgical trauma with excellent cosmetic results, both the patients and referring physicians are favoring MIAVR over the conventional procedure via median sternotomy. Indeed, various studies show equivalent or even superior outcomes of MIAVR compared to conventional surgery^[2]^. Additionally to the excellent cosmetic result, numerous studies report a decrease in blood transfusions, shorter intensive care unit (ICU) length of stay, and reduction in the rates of wound infections as well as postoperative pain after MIAVR, with no difference in postoperative mortality^[3]^. Furthermore, right anterior thoracotomy (RAT), with preservation of the sternum, has been proposed as an even less invasive method of MIAVR. Here, we describe our RAT approach using sutureless aortic valve prosthesis to achieve the best functional and cosmetic results without prolonging operative time. All patients consented for the surgery and use of their anonymized data for future research and publications. Data and materials are available for consultation. We also present our concept of simplifying preoperative imaging to achieve the optimal planning of the procedure.

## TECHNIQUE

### Preoperative Planning

Preoperative workup included diagnostic coronary angiogram, transthoracic echocardiogram, and a plain upright posterior-anterior and lateral radiographic chest film. A chest computer tomography (CT)-scan was only performed if the X-ray showed any pathologies or anatomical abnormalities to plan the incision.

Operative Technique

External defibrillator pads are placed on the back and left chest prior to draping. Transesophageal echocardiography (TEE) probe is placed for the evaluation throughout the surgery.

### Thoracotomy

We use a 6-7-cm incision to enter the chest through left anterior thoracotomy in the second to third intercostal space according to the surgeons’ assessment of the preoperative imaging. A soft tissue retractor is placed, and the ribs are spread with the retractor for additional exposure. We do not perform a rib-transection. The right internal mammary artery and vein are left untouched. After opening the pericardium, the stay sutures are placed to obtain an adequate exposure of the aorta. The stay sutures are brought out of the chest by the suture hook and tightened.

### Cannulation and Cardiopulmonary Bypass

**Fig. 1:**
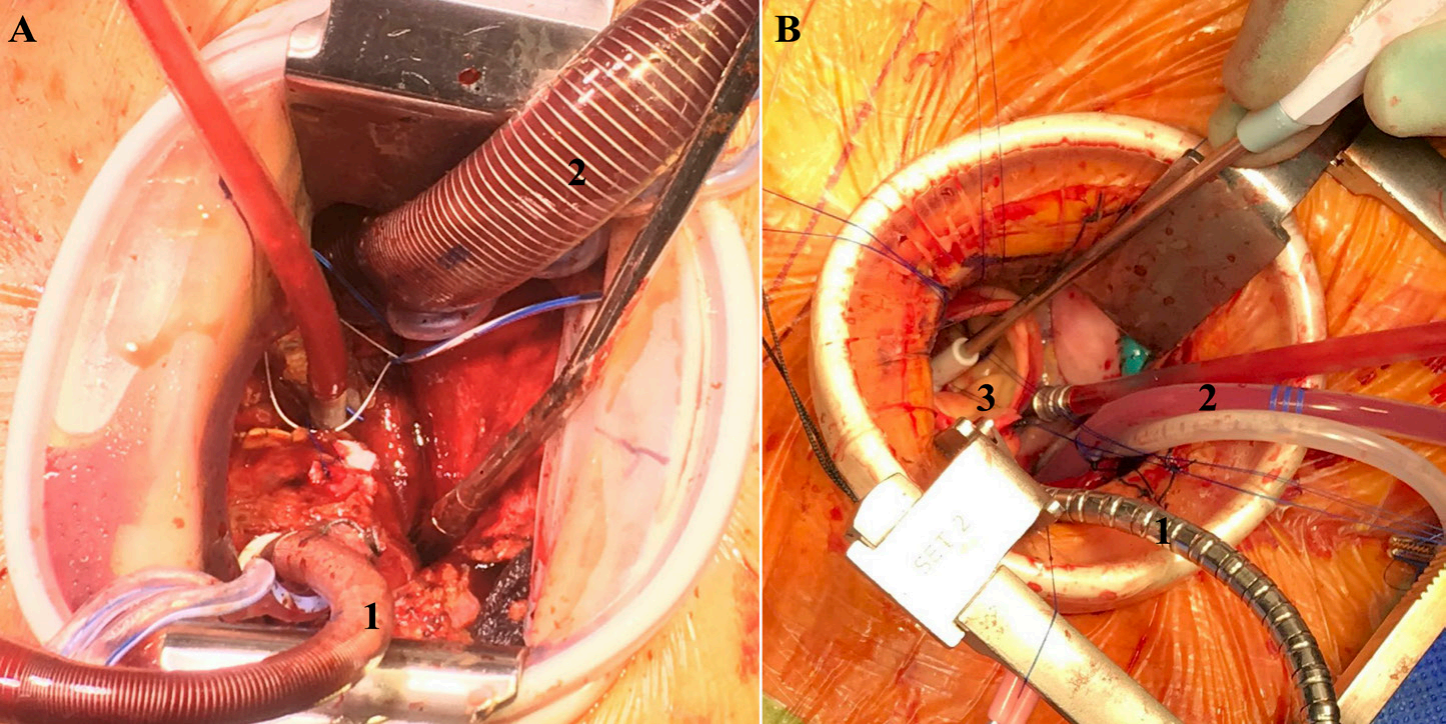
Right anterior thoracotomy approach, intraoperative situs after cannulation. A) 1, aortic cannula; 2, venous cannula; B) 1, aortic clamp; 2, left ventricular vent; 3, aortic valve.

We prefer direct cannulation of the aorta and right atrium with standard low-profile cannulas. If central cannulation is not possible due to the anatomical abnormalities or extensive calcification, peripheral percutaneous arterial and/or venous cannulation is performed with appropriately sized cannulas (Figures 1A and B). After establishing a cardiopulmonary bypass (CPB), a left ventricular vent is placed via right superior pulmonary vein in a regular fashion. The body temperature is maintained at 36ºC. Antegrade cardioplegia is applied via needle through the aortic root or selectively directly into the coronary ostia. Aorta is cross clamped using a flexible clamp. If the aorta is anatomically shifted to the left, a selective positive pressure ventilation with positive end-expiratory pressure > 20 mmHg of the left lung can be used to move the operating field to the right for the better exposure. This does not warrant selective intubation and can be easily achieved by a bronchus blocker.

### Aortic Valve Exposure and Replacement

**Fig. 2:**
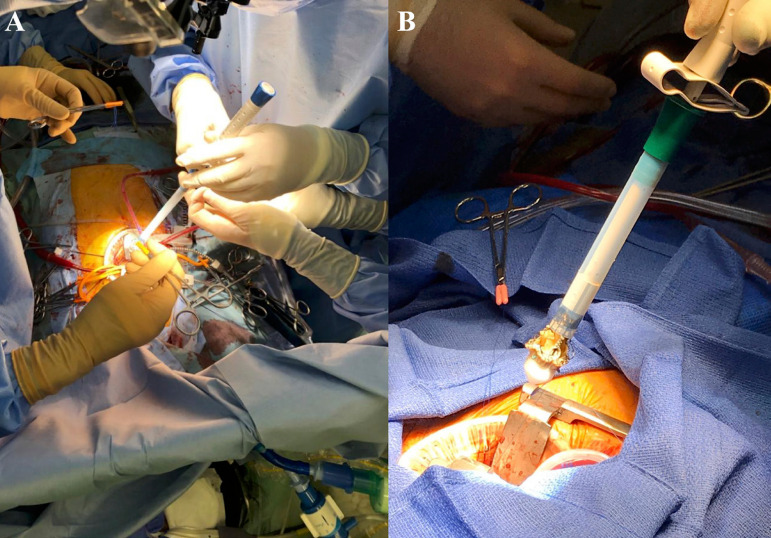
Sutureless valve implantation via right anterior thoracotomy approach. A) Bringing down the rapid-deployment valve system; B) rapid-deployment valve system ready for implantation.

A high transverse aortotomy is carried out to expose and assess the aortic valve. After excising and decalcifying the valve, the aortic annulus is sized with Perceval^®^ (LivaNova, London, United Kingdom) aortic valve sizers in a regular manner (Figures 2A and B). Then, rapid-deployment valve system is brought down into the position by the three 3/0 double-armed polypropylene guiding sutures, each one positioned into the corresponding nadir of the aortic sinus. After confirming the positioning, the valve is deployed in place as previously described by our team^[4]^. The prosthesis is checked again for proper anchoring and seat, and the aortotomy is closed in double layers.

A 32 F chest tube and pacer leads must be placed while on CBP and unloaded. Deairing is performed with TEE-guidance via aortic and left ventricular vent without direct manipulation of the heart. Separation from CPB and decannulation are performed. The valve prosthesis function is again assessed by the TEE, and the anticoagulation is reversed. The pericardium is partly closed to limit the adhesions of the lung to the heart or future possible procedures. A large suture is placed to approximate the ribs and the thoracotomy is closed in a routine fashion in layers.

## DISCUSSION

Over the last two decades, tendencies in surgical valve procedures are rapidly shifting towards minimally invasive strategies. In the growing era of transcatheter aortic valve implantation (TAVI), surgical procedures are being challenged by faster patients’ mobilization, shorter ICU and in-hospital length of stay, and less surgical trauma offered by TAVI.

Although MIAVR is often associated with longer operative and cross-clamping times, it has proven to be a feasible and safe procedure with lower postoperative mortality even in high-risk and elderly patients^[5,6]^. Indeed, not only does RAT maintain the sternum stability, providing the rapid mobilization, but it also prevents postoperative bleeding and provides lower blood transfusion rates and shorter ICU length of stay^[7]^. Cross-clamping time is known to be an independent predictor of morbidity and mortality in cardiac surgery. By combining the RAT approach with sutureless valve prostheses, we were able to drastically reduce the operative and cross-clamping times^[8]^. Through conventional central cannulation in most cases with low profile cannulas, we tend to avoid the groin complications associated with the cannulation of the groin vessels and longer immobilization.

During the preoperative planning, some groups^[9]^ use a chest CT to map the incision height. With our growing experience using RAT, we shifted from this concept to using echocardiography and X-ray to plan chest cavity access. CT-scan is performed only in the case of X-ray or echocardiography showing any abnormalities that could complicate the RAT access. This concept helps us spare time and reduce costs and radiation exposure.

**Fig. 3:**
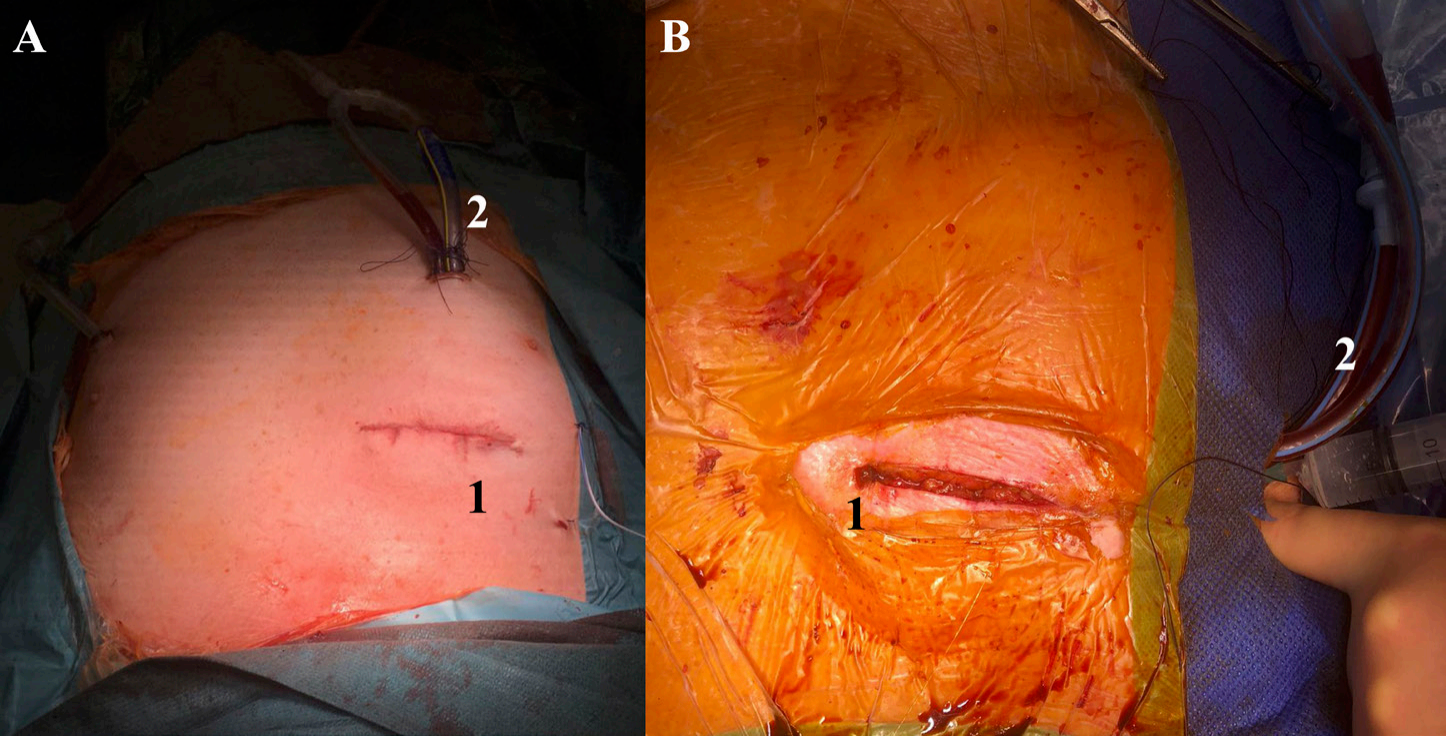
Cosmetic result of minimally invasive aortic valve replacement via right anterior thoracotomy approach. A) 1, 5-cm incision; 2, chest tube; B) 1, 5-cm incision; 2, chest tube.

Additionally, the RAT approach provides excellent cosmetic results with less pain and faster return to work, thus, improving patients’ satisfaction (Figures 3A and B).

Although surgeons have to go through the learning curve in order to perform MIAVR via the RAT approach, it can still be safely performed with an excellent hemodynamic profile and a low rate of postoperative complications.

In modern cardiac surgery, it is crucial to develop minimally invasive procedures. Based on our experience, RAT approach in combination with sutureless valve prosthesis provide excellent results. Operative and cross-clamping times, surgical trauma, blood transfusion, and ICU/in-hospital length of stay are kept low.

Preoperative imaging and careful planning are the key to success in MIAVR. Moreover, by using central cannulation, some groin complications can be easily avoided without impairing the surgical access to the valve. The superior cosmetic result provides higher patients’ and referring physicians’ satisfaction.

## CONCLUSION

Similar to any MIAVR, the RAT approach is technically more sophisticated compared to conventional surgical aortic valve replacement. Nevertheless, after completing the learning curve, there is no reason for surgeons not to provide this alternative method for their patients.

**Table:** 

Authors' roles & responsibilities	
AZ	Substantial contributions to the conception or design of the work; or the acquisition, analysis, or interpretation of data for the work; drafting the work or revising it critically for important intellectual content; agreement to be accountable for all aspects of the work in ensuring that questions related to the accuracy or integrity of any part of the work are appropriately investigated and resolved; final approval of the version to be published
KZ	Substantial contributions to the conception or design of the work; or the acquisition, analysis, or interpretation of data for the work; drafting the work or revising it critically for important intellectual content; agreement to be accountable for all aspects of the work in ensuring that questions related to the accuracy or integrity of any part of the work are appropriately investigated and resolved; final approval of the version to be published
BS	Substantial contributions to the conception of the work; agreement to be accountable for all aspects of the work; final approval of the version to be published
AAR	Substantial contributions to the conception of the work; agreement to be accountable for all aspects of the work; final approval of the version to be published
RV	Substantial contributions to the conception of the work; agreement to be accountable for all aspects of the work; final approval of the version to be published
DW	Substantial contributions to the conception of the work; agreement to be accountable for all aspects of the work; final approval of the version to be published
AR	Substantial contributions to the conception of the work; agreement to be accountable for all aspects of the work; final approval of the version to be published
AW	Agreement to be accountable for all aspects of the work in ensuring that questions related to the accuracy or integrity of any part of the work are appropriately investigated and resolved; final approval of the version to be published
